# Burkitt‐like lymphoma with 11q aberration

**DOI:** 10.1002/ccr3.2361

**Published:** 2019-08-06

**Authors:** Katrina Collins, Laila Mnayer, Peter Shen

**Affiliations:** ^1^ Department of Pathology Hartford Hospital Hartford CT USA

**Keywords:** aggressive non‐hodgkin lymphoma, atypical Burkitt, Burkitt, Burkitt‐like lymphoma with 11q aberration, cytogenetics and molecular genetics, WHO classification

## Abstract

Burkitt‐like lymphoma with 11q aberration is a recently recognized diagnostic entity in the Revised 4th Edition of the World Health Organization (WHO) classification of lymphoid neoplasms. This diagnosis should be considered and cytogenetic array performed in patients with high‐grade B‐cell non‐Hodgkin lymphomas without *MYC* rearrangement.

A 65‐year‐old man with multiple comorbidities presented with severe abdominal pain and rapidly growing mesenteric masses and bulky lymphadenopathy. Microscopic examination of the abdominal mass revealed large, pleomorphic cells with frequent apoptotic bodies and mitoses. By immunoperoxidase, the neoplastic cells show diffuse expression for CD20, CD43, BCL6, and c‐Myc along with very high proliferation rate by Ki‐67. BCL2 was negative (Figure 1). Very sparse CD3+ T cells are noted in the background. By flow cytometry, the abnormal cells were positive for CD19, CD20, CD10, and lambda‐restricted. Fluorescent in situ hybridization was performed using dual‐color break‐apart probes, and no *MYC*, *BCL2*, or *BCL6* gene rearrangements were detected. Moreover, by Affymetrix Oncoscan array, proximal gains and terminal losses in 11q along with the absence of 1q gain and extensive copy number variations were detected (Figure 1). These findings are diagnostic of Burkitt‐like lymphoma with 11q aberration.

**Figure 1:**
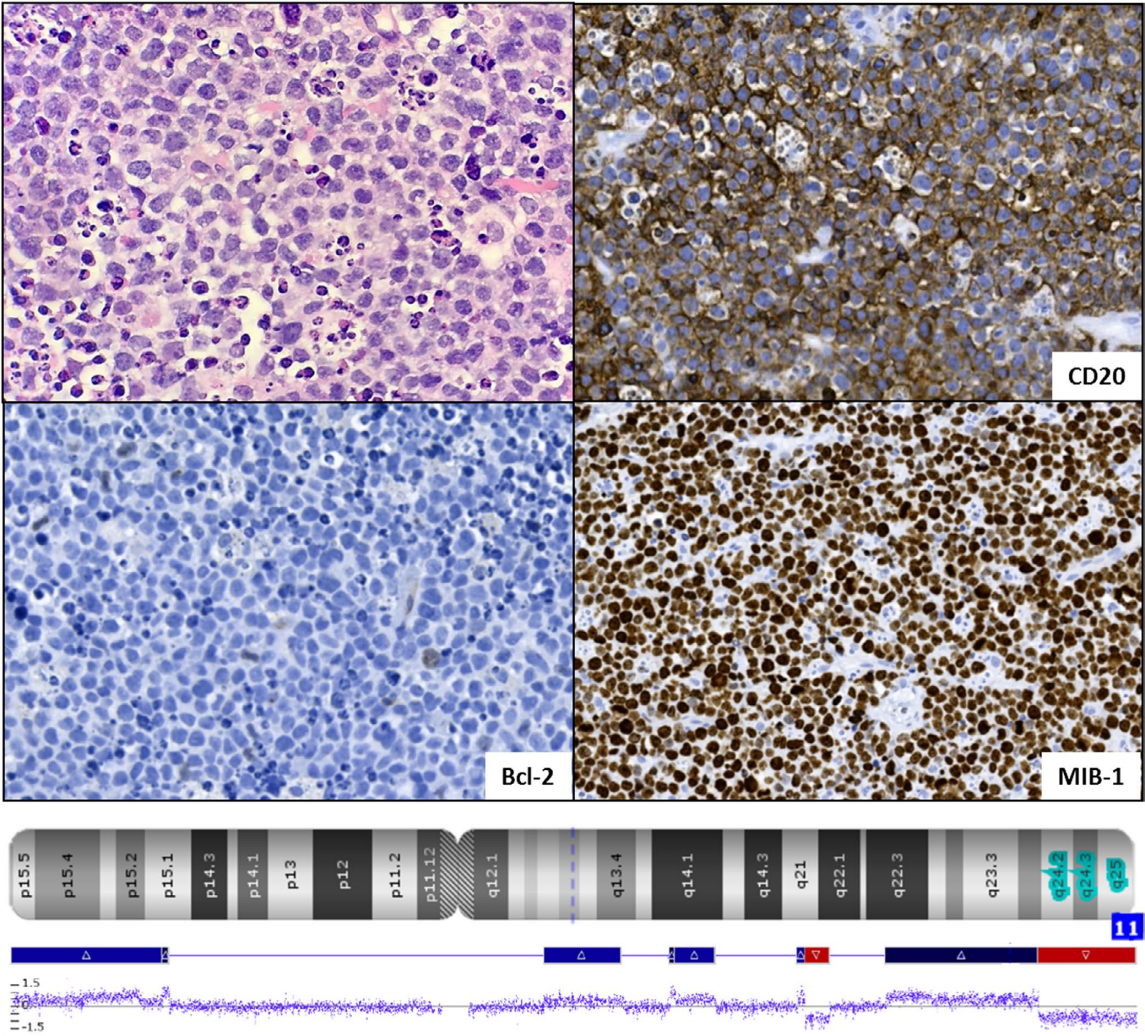
The tumor cells are medium‐sized to large, with diffuse expression of CD20, absence of Bcl‐2 expression, and high mitotic index. A starry‐sky pattern is seen. Chromosomal view of chromosome 11 analyzed by Affymetrix Oncoscan array, depicting gains of 11q21‐23.3 in blue and terminal losses of 11q23.3‐25 in red

Burkitt‐like lymphoma with 11q aberration is a recently recognized diagnostic entity in the Revised 4th Edition of the WHO classification of lymphoid neoplasms.^1^ This diagnosis should be considered and cytogenetic array performed in patients with high‐grade B‐cell non‐Hodgkin lymphomas without *MYC* rearrangement.^2^

## CONFLICT OF INTEREST

The Authors declare that there is no conflict of interest.

## AUTHOR CONTRIBUTIONS

KC, LM, and PS contributed equally to this manuscript.
